# Bibliology; or Analytical Reviews of Medical Publications

**Published:** 1818-10-01

**Authors:** 


### Second Department.

### BIBLIOLOGY:

OR

### ANALYTICAL REVIEWS OF MEDICAL PUBLICATIONS,

Nec tibi quid liceat sed quid fecisse decebit
Occurrat, mentemque domat respectus honesti.

CLAUD.

### Medicine.

### I.

*Results of an Investigation, respecting Epidemic and Pestilential Diseases; including Researches in the Levant, concerning Plague.* By CHARLES MACLEAN, M. D. Lecturer on Diseases of hot Climates to the Hon. East India Company. Vol. II. 8vo. pp. 524. London, 1818.

IF ever mortal enjoyed a heaven of happiness on this earth, that mortal must be Dr. Charles Maclean! Surely, that physcician must more than experience

"The soul's calm sunshine and the heart-felt joy,"

who not only believes, but is thoroughly convinced, that *he alone* has removed the veil from Nature's operations, and, by his *researches*, has laid open the means of "rescuing from destruction *several millions* of human beings *annually*." Preface, vi.

Independently of the heavy weight of guilt which we might incur by opposing such philanthropic plans, we should never forgive ourselves for disturbing the golden dreams of the Philanthropist himself, were we not assured from unquestionable evidence in the volume before us, that such reptiles in medical science as ourselves shall never be known, even by name, to the august Author; nor our sentiments honoured even with the most supercilious glance. Yet as we profess to have some twenty years personal experience of the subject here discussed; and as our opinions have already been favourably received by that profession of which we hold ourselves to be among the humblest members, we shall beg permission to take a slight, and but a very slight, notice of Dr. Maclean's omniscient doctrines and omnipotent practices. But, before proceeding farther, we shall state a circumstance or two, which will save a world of wonder in our Readers, and which we did not find out till we got to the end of the volume.

It appears, that in the year 1793, Dr. Maclean experienced a Coup de Soleil in his own person, which he treated solely with *mercury* (for the Doctor never bleeds in *any* disease), and that three years afterwards he published some Tracts at Calcutta. Now, gentle reader, how were these tracts received by his professional brethren in Bengal? Let himself tell.

"These publications procured me the distinction of being heartily reviled by those, who considered themselves as medical authorities in the East; who, instead of attempting to refute my arguments, *discreetly*, contented themselves with representing me as *a madman*, and calling me names." P. 505.

This is a mortifying confession; and we should here have dropped the pen, and allowed the work to glide silently to that land of "shadows, clouds, and darkness" to which it is rapidly hastening, did we not conceive, that as a public Teacher, some of his doctrines and practices might, however absurd or erroneous, adhere to the juvenile mind, whose inexperience wavers between contrasting authorities.

We shall not follow Dr. Maclean through his romantic adventures near the "Seven Towers," or hair-breadth escapes in the Levant. These are related with all due regard to stage effect; and it is only when pestilence is touched upon, that the cloud of false hypothesis wraps every thing in impenetrable darkness, and snatches from our sight the lively and entertaining traveller.

As a Brunonian, Dr. Maclean "out-herods Herod," and leaves the memorable advocate of brandy and laudanum far behind.

"The nature of all morbid affections is the same, by whatever remote cause they may be produced.* Epidemic, *like other diseases*, consist of *diminished* excitement of one, or more, or several,

---

* "Elements of Medicine. These "Elements," by which all things are proved in these volumes, *are not yet published!* This is a way, with a vengeance, of refuting the arguments of an adversary!

organs of the living body, in all the combinations, proportions, and degrees in which they can be affected by a noxious atmosphere, and other concurring causes." P. 388.

This illustrious physician admits of no such thing as *increased* excitement in any disease; the most intense phrenitis or pneumonia is a disease of "diminished excitement," and consequently requires stimulants!

The melancholy effects of the "insolation," before alluded to, are, if possible, more conspicuous in the therapeutics than in the pathology of this volume.

"Amongst the most pernicious of the numerous errors which, upon the mere ground of hypotheses, have pervaded medicine, almost from the origin of Societies, is *the deleterious practice of blood-letting.* This species of evacuation, *although injurious in all diseases,* is, in its consequences, pre-eminently destructive in those which are epidemic and pestilential. That blood-letting is an operation which ought never, *under any circumstance, or in any situation,* to be performed, I have shewn in this volume by a great variety of proofs. My observations, *although hitherto not published,* as a whole, were almost all written a great many years ago, chiefly in the East Indies." xi.

We will not insult the ears or the understandings of our readers by quoting another passage, or making another comment. We shall conclude this article with a prayer as fervent as any that ever issued from our lips—a prayer with which we recommend our Author to conclude each of his "Lectures on the Diseases of hot Climates:" May God, in his infinite goodness and mercy, have compassion on the *souls* (for the *bodies* are beyond redemption) of such of our countrymen, in the East and West Indies, as come under the doctrines or practices inculcated in these Volumes!

P. S. We had almost forgotten to announce an important discovery of Dr. Maclean's, namely, that ptyalism is not caused by *taking* mercury, but by *leaving it off;* and consequently, that we may, at any time, prevent this disagreeable accident *by persisting* in the administration of the medicine!

"Ulcerations of the throat, soreness of the mouth, *salivation,* purging, &c. arise, not from the immediate action of mercury, but from its irregular application, or *sudden subduction.* If any one affects to doubt this fact, let him take one grain of calomel every hour, for twenty or thirty hours, and then stop. He will find that his mouth does not become sore while he is taking the calomel at regular periods, but some hours after he has left it off; that the soreness will continue to increase for some time after having desisted from taking the medicine, *and may be diminished or removed* by a proper re-application of the same power." P. 500.

### II.

***Report of certain morbid Conditions of the Abdominal Viscera in some Varieties of Maniacal Disease, with the Methods of curative Treatment; and Observations on Epilepsy.*** By Dr. Ed. Percival, of Dublin.

[Dublin Hospital Reports, vol. i.]

As we do not restrain ourselves within any other limits than those of *practical utility*, we shall very often step aside from the beaten track, and single out an individual article, from a miscellaneous volume, for the subject of an analytical memoir, convinced that by so doing, we more effectually obtain the great object of our labours—the diffusion of practical knowledge.

The records of insanity are remarkably defective of information respecting the morbid condition of the abdominal organs in that disease. Although the ancients discerned the connexion between the viscera and the *brain;* yet the moderns have almost exclusively directed their pathological attention to the *latter*, and not with much success, as may be learnt from the confessions of Haller, Pinel, and Esquirol. The organs of digestion hold a manifest sympathy with the cerebral structure, whose morbid conditions are variously associated with derangement of the mind. The disorders of these lower viscera are also much more cognizable, both in the living and dead subject, than those of the higher organ, and they are more accessible to medical management. *Moral* means are no doubt important auxiliaries; but the labours of pathology must not be sacrificed to the light suggestions of scepticism.

Pinel only re-asserts the doctrine of Areteus, when he says, that "the primary seat of mental alienation is in the stomach and intestines, from which centre it propagates itself, as it were by irradiation, and deranges the understanding." Spurzheim remarks, that "the greatest number of cases of insanity produced by sympathetic causes, originate from deranged functions of the digestive organs." 300.

*Intestinal Torpor, with increased Secretion from the mucous Membrane.* Mania is frequently ushered in by an acute and febrile paroxysm, and the patient exhibits a dusky, suffused complexion, shining eye, and *fœtid breath,* denoting disordered function of the stomach and bowels.

The tremulous tongue is often coated with mucus; the appetite is impaired or depraved; there are intestinal heats and thirst; the heart labours, and beats powerfully against the ribs; the carotids throb; the bowels are constipated; urine scanty, depositing sediment, and offensive in its odour. 'Nothing is more remarkable than the fœtor which taints the atmosphere of the patient, and which is most offensive when the constipation has been of longest duration. Dr. P. has observed a similar exhalation in three cases of catalepsy with obstinate constipation. We ourselves have often remarked a *stercoraceous* odour from the *perspiration* of constipated patients. The same may be observed in the remittent fever of children. In maniacal cadavers, the serous effusion of the brain emits this peculiar odour. If the abdomen be examined in an acute paroxysm, it will be found tumid, especially about the epigastrium. In some, there is great sensibility to pressure about the hepatic region.

When medical aid is neglected, the symptoms are exasperated; perfect anorexia, inextinguishable thirst, foaming at the mouth, florid countenance, intestinal ardour, fever, insomnium, &c. supervene. If medicine be inadequate, Nature generally brings relief by some evacuation. *Hæmorrhage* affords a precarious and transient relief; vomiting is still worse; perspirations are almost nugatory. The true critical and salutary evacuation is from the bowels. A spontaneous diarrhœa supervenes suddenly, the dejections are frequent, involuntary, and copious.

"If the unassisted efforts of Nature thus evacuate fæcal and mucous excrement, in consistent masses, great, and often decisive relief is obtained by the patient. But it more commonly happens, that the dejections are thin and acrid; the fluids taken into the stomach passing through the canal with little alteration, further than being impregnated with some morbid secretions. Under these circumstances, the diarrhœa is slowly converted into dysentery of the milder kind; the patient's strength and flesh waste rapidly, and if he is not seasonably relieved by judicious treatment, he falls into an incurable hectic atrophy. During this process the character of his insanity changes from violent excitation to more tranquil delirium—from restlessness to quiescence; at length to torpor, amentia, and insensibility."

M. Pinel calculates from extensive data that *three-eighths* of the fatal cases of insanity terminate in diseases of the viscera.

To restore the healthy functions of the digestive organs in these circumstances is no easy matter. The throbbing pulse, turgid countenance, suffused eye, and depraved secretions, are evidences of unequl vascular excitement and sub-inflammatory condition of some parts of the system, which, in young or robust subjects, demand the lancet. In such cases, it is difficult to procure catharsis, and hazardous to prescribe an emetic "*without premising venesection.*" But the first blood-letting should not exceed fourteen ounces; for maniacal excitement quickly veers round to debility and exhaustion. After V. S. a combination of emetic and purgative medicine answers best—instance, two grains of calomel and half a grain of tartrite of antimony may be given every hour until vomiting is excited. If catharsis do not follow, senna, salts, and tincture of jalap may be administered at short intervals, until the bowels are well evacuted. Sometimes a large draught of cold water determines the bowels at once to action. This we have repeatedly experienced in our own persons, and witnessed in others. After much fæculent matter, a gelatinous substance is expelled, especially in hypercatharsis or dysentery, of a dull greyish hue, and perfectly viscid. It often brings relief to the febrile symptoms, and removes the peculiar fœtor already mentioned. It is discharged copiously from the stomach by emetics, and the patient is often incessantly employed in disengaging a similar secretion from his fauces, and spitting it all around him; this is one of the most certain evidences, (though little noticed) of the general condition of the mucous surfaces here decribed. This extraneous sheath itself must render the intestinal canal insensible to the stimulation of food or physic. But there is an *intestinal torpor,* like that in hydrencephalus, much greater than can be explained by this mechanical cause. Dr. P. well observes, that

—"It is rather to be ascribed to inertness of the peristaltic energy, and to that *disturbed balance* of the visceral functions, which co-operate with disordered secretion, to impede the whole process of digestion, assimilation, and discharge." P. 130.

Here several grains of emetic tartar are necessary to excite vomiting; if calomel be combined with it, catharsis is more certain to follow. The concussion which the whole intestinal canal undergoes by these conjoint operations, speedily eliminates the mucous sordes, with other fæculent materials, in prodigious quantities, with great and evident relief.

Although the mental malady returns with little diminution, yet some advantage is always gained in this way. The train of disordered action is broken for a time, and some of the sources of mental irritation are removed. After a lapse of twenty-four hours, bleeding may be again necessary, as indicated by the throbbing celerity of the pulse, manifesting a condition of the vascular system inimical to healthy action of the secreting organs. It may be termed a sub-inflammatory state. After venesection, a mercurial purgative may be given, remembering that the former facilitates the operation of the latter. A copious discharge of bile generally ensues, with some appetency for food, and inclination to sleep. Concurrently with these, the head should be shaved, and sponged with cold or tepid vinegar, iced water, or æther. Blisters at an early stage, or anterior to evacuations, are injurious. Fomentations to the legs and feet are often useful.

As an alterative of the digestive organs, Dr. P. has found the pilula hydrarg. in combination with ipecacuan, a highly serviceable medicine. Two grains of each may be given twice a day; and should the bowels become too relaxed, four grains of Dover's powder may be substituted for the ipecacuan. If the urinary discharge be deficient, taraxacum, with or without the squill, may be advantageously given.

Dr. P. truly observes, that in every disorder, local or general, it is of the utmost importance to restore and preserve the equilibrium of the functions throughout the system; nor is this principle less applicable to mental derangement than to other diseases. The most judicious treatment cannot restrain the fixed course of the small-pox, measles, or scarlet fever; yet no one entertains a doubt of the invaluable resources of the medical art in conducting those disorders to a favourable issue. And so it is with insanity; although the practitioner is often foiled in his efforts to restore reason as speedily as he could wish, yet he should not remit his diligence for a single day in maintaining the best possible condition of the bodily functions. By attention to the digestive organs alone, the painful irritations of hunger, thirst, or anorexia, may be mitigated or removed; nutrition, strength, and cheerfulness, may also be considerably supplied; the patient becomes more tranquil and amenable, and derives from the comforts of food and sleep considerable appeasement of mental irritation.

Dr. P. is of opinion, that *extreme* venesection is to be deprecated in mania.

"The result of this practice has been, in some cases, to convert deep melancholy into fury; in others, to precipitate phrenzy into incurable imbecility both of body and mind." P. 137.

He has not found that bleeding from the jugular vein possesses any superiority over that from the arm. Moderate blood-letting, at intervals, is highly salutary in a great proportion of acute cases.

The fatal apoplexies supervening on the operation of emetics, may generally be referred to the omission or scantiness of blood-letting, previous to such discipline. That fulness of the vascular system of the head, evinced by stupor or delirium, head-ache, red eyes, &c. is always relieved by venesection; and so is that congestion of the abdominal viscera characterized by deficient secretion, local tenderness, and quickened circulation.

Inappetency for food, coated tongue, and slow bowels, indicate the use of emetics. The nausea increases the exhalation of thin fluid from the mucous membranes, which serves first to detach, and then to facilitate the discharge of the more tenacious material adhering to their surfaces. The biliary, pancreatic, and gastric glands, are, at the same time, emulged, and the irritation excited by congestion there is thus removed.

*Diarrhœa.* In chronic mania, the bowels are more prone to diarrhœa from cold, or errors in diet, than in sound health. This is particularly the case in scrofulous subjects, and more frequent in idiots than in maniacs. The complaint is often owing to mismanagement of the torpor in the first or acute stage of the disease. It requires cautious treatment. If attended by irritative fever, a moderate venesection should be premised. The pulv. hydrarg. c. creta, combined at first with rhubarb, and afterwards with Dover's powder, is a useful remedy. A pill, composed of opium, ipecacuan, pil. hydrarg. and carb. ammon. has also proved efficacious. The warm bath, an hour every day, is a powerful auxiliary.

On dissection of these patients, the intestines generally exhibit an extensive mass of disease. The mesenteric glands are enlarged and indurated; the mucous membrane inflamed, thickened, and partially eroded, and the area of the canal diminished, often considerably in the lower intestines.

*Voracious and depraved Appetite.* Two different and opposite affections of the stomach frequently occur in mental derangement—bulimia and anorexia.

A *voracious appetite,* unattended with marasmus, is not a dangerous symptom, but too often indicates confirmed insanity tending towards fatuity. That in these cases the secreted fluids are acescent in their quality, is proved by the relief which magnesia, lime-water, and the alkalies afford: but these agents being chiefly chemical, their benefit is often transitory, and the practitioner is obliged to consult the further resources of his art. A vomit will often allay this irritation for many days. Nauseating medicines will also suspend, *pro tempore,* the voracity. Small doses of opium or cicuta, combined with the blue pill, are useful in controling or amending the vitiated secretions, and so is milk with lime-water. The food of the patient should be substantial, and distributed at equal intervals through the day.

*Aversion to food.* This is not uncommon; is difficult to manage, and hazardous in its consequences. It has its source, in most instances, in visceral disorder. Abstinence alone will produce anorexia, and the desire for food ceases altogether, after a certain period, the pain of hunger being lost in consequent debility, fever, and delirium. Mild mercurial purgatives, the warm bath, frequent injections, a light but nutritive diet, are the chief resources in this emergency. If phrenitic symptoms appear, blood-letting and cold applications to the shaven head will be indispensable. If cataleptic appearances should come on, venesection is also requisite, with boluses of calomel and scammony.

It does not appear from dissections, that lunatics are prone to scirrhus of the stomach or pylorus.

"But they are undoubtedly prone to hepatic disease of various kinds; and hence often arise incurable disorders of the primæ viæ. Chronic anorexia, so far as I have observed it, is always attended with torpor of the liver, indicated by pale and scanty fæces, cadaverous complexion, and inert bowels."

Under these circumstances, the flesh wastes, and a confirmed aversion to food is soon established.

### MANIACAL EPILEPSY.

A deplorable species of Insanity supervenes on certain cases of Epilepsy. It is of the chronic and periodical kind, tending more or less rapidly to fatuity. Although these patients are generally deemed incurable, their maladies admit of alleviation. In the year 1813, Dr. P. made trial of the rectified oil of turpentine in epilepsy, and published the results in the 9th volume of the Edinburgh Journal. Since that time he has had numerous proofs of its salutary influence in the disease; so much so, as to satisfy him that it is incomparably the most efficacious remedy that we are yet acquainted with for that intractable disorder. He has made extensive application of it in the epileptic cases of the Hardwicke Lunatic Asylum. All of these cases had been of long standing, and deemed incurable; so that alleviation of suffering was all that could be hoped for. He was not able, in a single instance, to banish permanently the epileptic attacks; but in every instance they became considerably milder, less frequent, and remarkably disengaged from the maniacal excitement which had formerly attended them.

Dr. P. details seven out of twenty cases of epilepsy treated with oil of turpentine. The following is the form in which he usually prescribed it. An ounce of the oil was triturated with an equal weight of lump sugar, and a pint of spearmint water was gradually added. A full dose of the emulsion is an ounce three times a day. The salutary effects of this medicine in the cases detailed are very evident; for, besides the striking alleviation of their epileptic disorder, and its maniacal adjuncts, all of them gained considerable improvement to their general health, appetite, and cheerfulness. It may be remarked, that women bore larger doses of turpentine, without experiencing catharsis, than men. In them too it generally proved soporific, which was not the case in the male sex. Dr. P. does not seem to be acquainted with the effects of the lytta in epilepsy; but the present number of our Journal will probably lead the attention of the faculty to this important remedy.

The full delineation which we have given of this interesting paper of Dr. Percival's, precludes the necessity of any comment from us. Accuracy of judgment, keenness of discrimination, and modesty in opinion, are among the prominent traits of this gentleman's writings:—these are the surest passports to *public* approbation, as they are the essential requisites in *private* practice.

### III.

*Practical Illustrations of Typhus Fever, and other Febrile and Inflammatory Diseases.* By JOHN ARMSTRONG, M. D. *Second Edition.* London, 1818.

IN our review of the First Edition of this work we confidently predicted the favourable reception it would meet in the medical world. These predictions have been fulfilled. The present Edition is considerably improved. The subject of typhus itself is re-arranged under heads, which is advantageous. The section on Dysentery is enlarged, and that on Mania greatly. As this is a subject on which we did not touch in our analysis of the First Edition, we shall select it for the present article, as a proper sequel to that on the same disease from the pen of Dr. Percival.

### MANIA.

Dr. Armstrong considers that this disease may be a primary affection of the brain, or secondarily induced there by distant disorder, as of the digestive organs. Like apoplexy, it is either acute or chronic, and also marked by venous congestion or arterial excitement. Affection of the liver, our author believes, and in this we agree with him, as often follows as precedes the cerebral affection, appearing sometimes as cause, and sometimes as effect.

"When an affection of the liver, or indeed of any other remote organ, operates on the brain, so as to produce ultimate madness, this operation is not direct but indirect; for the affection of the remote organ often proves an irritant to the heart, the increased action of which excites mania, by propelling the blood too powerfully towards the brain. Or the return of venous blood being interrupted through the remote organ, as may happen in cases of congestion of the liver, the circulation of the brain is thereby mechanically affected, and madness succeeds." P. 288.

This view of mania, Dr. Armstrong justly observes, presupposes an antecedent disposition in the brain to morbid action, which may exist under ordinary states, without producing disturbance; yet when the system receives a shock, the effects of that shock will be concentrated in the weakest part.

"What we call increased determinations of blood to particular parts are, in general, merely obstructions in the smaller vessels, by reason of which the blood cannot be so readily returned through the veins; so that the currents of blood transmitted by the heart through the larger arteries, continue to accumulate in those arteries, which accordingly become more distended than others. Local determinations of blood, as above explained, are very conspicuous in many diseases, but in none more so than in mania." P. 298.

The acute attacks of mania, congestive as well as excitive, are often so formidable as to threaten immediate danger from venous congestion or arterial excitement, requiring at their commencement a treatment similar to that of congestive or excitive apoplexy.

Sudden chills, the depressing passions, sedentary employment, and indigestible food, often lead to the congestive variety; while exposures to a high temperature, strong mental emotions, stimulating drinks, and full diet, lead to the excitive variety. It has been a bitter national reproach, that one half of the population is scrofulous and the other mad! Our author conceives that there is more affinity between the two diseases than is generally imagined. Scrofula and mania are frequently produced by the vicissitudes of our climate, and the full and irregular diet which we use, together with wine and spirits. Here Dr. Armstrong takes occasion to lash the intemperate habits of the upper classes of society, which spread among the lower, and produce infinite mischief. This subject will shortly be brought before the public in a striking point of view.

Dr. A. makes many excellent remarks on the chronic forms of mania, which steal on insidiously for a long time before the subjects of them can be pronounced positively insane. Dejection of the spirits is usually the precursory symptom, followed by defect of memory, dullness of intellect, and change in the expression of countenance.

The *excitive* form of chronic mania is generally preceded by watchfulness or disturbed sleep; uneasiness in the head; quickness and firmness in the pulse; whiteness of the tongue, and slight increase of heat at nights. Irritability next succeeds, with change in the demeanour, or even in the moral character. Although the liver is often in fault, Dr. A. thinks that it is more frequently affected consecutively than primarily. Here our author contends warmly for the immateriality of mind, and the corporeal nature and seat of insanity. Hence, to remove mania, we must restore to a sound condition the organ of the mind. The disease our author considers remediable in its incipient stages; incurable when it has existed for some time; and on this principle, that at *first* it depends on disordered action, *afterwards* on change of structure, or at least a morbid state of the vessels of the brain.

Dr. A. never found the brain of a maniac perfectly sound after death.

In incipient insanity, with great fulness of the vessels, depletion, both by bleeding and purging, must be quickly effected, and a perseverance in this plan for some time will generally be necessary, combined with a regular course of mercurials. After the pressure of the congestion or excitement has been evidently mitigated to a considerable extent, depletion by the lancet should be cautiously pursued, since, if carried too far, it may occasion a nervous irritation that acts powerfully on the heart, and may tend to protract the disease. In the first two weeks of the attack, our author bled both from the arm and temporal artery, until the fulness of the cerebral vessels was relieved, and the action of the heart restored to a more natural state, the bowels being kept well open with calomel, jalap, and the sulph. mag. the calomel being given in full doses, to procure its alterative effects as well as its purgative. After that period, he was accustomed to draw blood by leeches, or cupping, about twice a week, with a saline purgative every second morning, and to give calomel daily, so as to insure a moderate but constant ptyalism for some time. At least two thirds of the cases of recent madness which our author attended, recovered within the first three months, under this treatment, though hardly any of the patients shewed signs of convalescence until the mouth had been affected for some weeks by the mercury, and until some degree of emaciation had taken place. This remark, and this whole division of the work, is highly deserving of attention.

We cannot pursue the subject further, and have only room to observe, that the present edition is greatly enlarged and improved in most points, but especially on the one just closed, as well as on rheumatism, delirium tremens, apoplexy, and inflammation of the viscera.

In the Fever Institution, to which Dr. Armstrong has been recently appointed, he will have ample scope for the exercise of those pre-eminent talents which the public has acknowledged, and his professional friends know him to possess. May the sun of science cast a meridian splendour on his investigations! May the great, the good, and the wise, encourage a man whose life is dedicated to the improvement of the noblest of arts—that of mitigating human sufferings, and preserving human health!

### IV.

### *Case of Melena; with Observations on the alternate Excess of Morbid Action in the mucous and serous Membranes.*

By J. Cheyne, M. D.

[ Analyzed from the Dublin Hospital Reports, Vol. I. ]

Case. Michael Quilty, ætat. 24, was admitted into the Meath Hospital, January 10, 1812, in a state of great debility and exhaustion: face bloodless; eyes languid; features relaxed; moaning and shivering; breath pungent like that of a drunkard, but more carneous; heat of the body increased; extremities cold. Pain in the lower part of the epigastric, and upper part of the umbilical regions; pulse 120; tongue dry, pale, and coated; legs, along the shin bone affected with a vesicular eruption, containing a yellowish lymph.

The preceding day, nausea and pain in the upper part of the abdomen were succeeded by a discharge, upwards and downwards, of black matter and clotted blood, to the amount of four or five quarts. The hæmorrhage returned at intervals during the night. *A blister to the abdomen; a drachm of sulph. magnes. every hour in mint water.* 11th. He continued to pass by stool a quantity of inky matter intermixed with blood, and is becoming weaker and weaker. Small doses of the ol. terebinth. every two hours, which stopped the sanguineous discharge, but constipated the bowels for three days, during which the abdomen swelled. 16th. A fluid evidently in the abdomen, and the stools when at last obtained, were hard and colourless. 18th. Pulse 96, urine scanty. Crystals of tartar; blue pill and squills; urine increased, but no change in the stools. In two days the gums were affected. 28th. No bile appeared in the stools after purgatives; urine free, pulse 108. Hard dry cough, mouth sore: evening a return of the hæmorrhage; four large basons full of blood were discharged from the stomach, and about a pint of pitchy matter from the bowels. 29th. Pulse 136; countenauce pale; voice feeble; less tension of the abdomen; hæmorrhage from the stomach and bowels, about a quart; soreness of the epigastrium. 30th. Distension of abdomen gone; lost another quart of blood, and died at five o'clock.

*Sectio Cadaveris.* No evidence of swelling in the abdomen externally; not more than three pints of fluid in the cavity; omentum loaded with fat, as was the mesentery. The *serous* membrane lining the whole of the membranous viscera was exanguious. Over the *mucous* membrane of the stomach, there was a general blush of inflammation, particularly round the cardiac orifice; round the pyloric extremity a blackish mottled appearance, as if from extravasation; many white opake spots all over the internal surface of the stomach, which was coated with a very viscid mucus that was with difficulty scraped off, and appeared tinged with blood. The condition of the duodenum, jejunum, and ileum resembled that of the stomach. Some pitchy matter in the ileum towards its extremity. The same phlogosis on the mucous membrane of the colon; no fæculent matter in any part of the intestinal canal; liver indurated, its left lobe exceedingly enlarged, filling the epigastrium, and dense throughout the whole substance, its vessels containing no blood. The spleen was double the usual size, its serous membrane thickened and opake. About a quart of yellow fluid in the cavity of the thorax. Heart and all the great vessels exanguious.

Dr. Cheyne candidly remarks, that the turpentine not merely suspended the hæmorrhagic effort, but the *natural secretions* also, of the intestinal canal; the consequence of which was an effusion into the cavity of the peritonæum, In the course of five days, fluctuation was manifest. The mercurial, as is usual after great losses of blood, soon affected the system. In this state the powder of jalap and squills reproduced the hæmorrhage in a fatal degree. But no sooner was the mucous surface of the intestines again excited, than the excitement of the serous exhalents subsided; the bulk of the abdomen was speedily reduced, and upon dissection, there were found not more than two or three pints of fluid in a cavity which, a few days before, must have contained, at a moderate calculation, seven or eight quarts; in fact, the case then formed a well-marked one of ascites. The "*Medicine expectante*" might, our author modestly thinks, have been more felicitous. The case, however, is very instructive in a pathological point of view, and it drew the attention of this eminent physician afterwards, to the subject of *alternate* excess of morbid activity of the mucous and serous surfaces—a point which will be further illustrated by the following case.

A postillion, ætat. 18, was admitted into the Meath Hospital, the 8th of October with ascites. Abdomen very prominent and tense; skin dry; urine scanty, depositing a pink sediment; tongue furred; thirst; general health not much impaired.

Three weeks before admittance, he was attacked with rigors followed by frequent inclination to stool, with tenesmus, discharges of slime, and occasionally hardened fæces. At this time, and previously, he was exposed to wet, and cold feet. The bowel complaint continued nearly a fortnight, when it suddenly stopped, and he began to perceive a fullness of his belly, which progressively increased. While in the hospital he had several attacks of severe pain in the abdomen, followed by tenderness and impatience of pressure between the umbilicus and the pubes. They were relieved by anodynes, and prevented by local blood-letting. The blue pill with ipecacuan; castor oil. 17th. Gums sore; tension of the abdomen increased; urine still insufficient in quantity. Omit the mercurial. Crystals of tartar and jalap, with sp. eth. nit. 21st. Urine increased; belly subsiding fast. 28th. Head-ache and oppression of the stomach, with white tongue and quick pulse. A mild mercurial again ordered. 31st. Urine copious; belly quite reduced.

November 3d. Mouth again sore; discontinued the mercurial; and discharged the 20th of November.

This case, Dr. C. thinks, was a striking instance of the transference of disease from the mucous to the serous membrane of the abdomen. On the sudden cessation of dysentery, the abdomen began to fill with fluid, in consequence of the inflammatory excitement of the peritoneum. There were four inflammatory attacks, each being succeeded by a dropsical effusion with increase of bulk. This inflammation was subdued by topical applications, and then the mucous membrane of the intestines being stimulated to greater secretion by crystals of tartar and jalap, the dropsy quickly disappeared, and the patient recovered. "Anasarca, as well as dropsy of the cavities, seems to be connected with an inflammatory excitement of these membranes." It would be no difficult matter, our author thinks, to shew the interchange of morbid activity in the vessels of the serous and mucous membranes of the thorax. How often are the premonitory symptoms of hydrothorax relieved by free expectoration, and *vice versa*.

"In many cases of hydrothorax, the best anti-hydropics are, *first*, such medicines as have a tendency *to reduce* increased action in the pleura, namely, digitalis, and occasionally bleeding. *Se-*

*condly*, such remedies as produce excess of action in the skin, as blisters; and, *thirdly*, such diuretics as have also the power of increasing expectoration, as squills." P. 271.

We shall have repeated occasions of illustrating these doctrines of Dr. Cheyne by facts noticed by our continental brethren, who, in our opinion, have taken the lead of us in many points of pathology, but especially in the doctrine of metastasis of action from one tissue or series of tissues to another. Meantime, we return our thanks to Dr. Cheyne for the interesting practical views laid open in this paper.

---

### V.

*Observations on the Properties of the Air-Pump Vapour-Bath, pointing out their Efficacy in the Cure of Gout, Rheumatism, Palsy, &c. &c. &c.* By M. La Beaume, Medical Electrician. Octavo, pp. 84. London, no date.

This Pamphlet is not addressed to the Profession. It is all directed to the *ends*, and contains nothing of the *means*. The sum total of information is this, that Medea's cauldron is boiling in Southampton Row, where every "ill that flesh (and even bone) is heir to," may be removed by the air-pump, the vapour-bath, and medical electricity! The old story of fifteen pounds pressure per inch upon the surface is again reiterated, and Dr. Blegborough's papers on the Air-Pump Vapour Bath are here republished. Galvanism is henceforth to be the Diacatholicon; and M. La Beaume, by his wonderful talents and apparatus, can now pour "the soul of the universe" into the great toe of an invalid! "I feel justified, says he, in asserting, that the whole Materia Medica may be searched in vain, to find such a PANACEA as the galvanic influence, &c. P. 77.

But it is against MERCURY that this distinguished philanthropist declaims with the greatest eloquence. The "nervous temperament" of Trotter, and the "digestive organs" of Abernethy, are held up in terrorem to frighten the mercurialists from the field; and our author boasts of the patronage — "The friendly aid and kind services," of Drs. Bateman, Laird, Roget, Sims, Southey, Buchan, Davis, Scudamore; and of Messrs. Cooper, Pearson, Blair, Sir William Adams, Maule, Ogle, &c. &c. But *fronti nulla fides*; we all remember a celebrated "Guide for Tropical Climates," ushered in under the *express auspices*

*and approbation* of Dr. William Dick,' who disclaimed all knowledge of this liberty, the moment he saw the production. We can hardly think that the abovementioned distinguished ornaments of the profession ever lent their "friendly aid and kind service" to such heterogeneous compositions as the present pamphlet contains.

---

### VI.

*A Treatise on the Nature and Cure of Gout and Rheumatism, including general Considerations on Morbid States of the Digestive Organs; some Remarks on Regimen; and Practical Observations on Gravel.* By CHARLES SCUDAMORE, M.D. Member of the Royal College of Physicians, &c. The Second Edition, corrected and much enlarged. Octavo, pp. 592. London, 1817.

It was our intention to have presented an elaborate analysis of this most valuable and masterly production; but so extensive has already become the circulation of the work, and so justly have its merits been appreciated both by the profession and the public, that we deem such labour no longer necessary. For miserably contracted and groveling must be the views and principles of the man; who, calculating upon the economy of a little time and money, would prefer the scanty information afforded by the most luminous abstract that could be written, to the rich and ample store contained in the original of a work like this. With equal profit and consistency would the thirsty traveller halt contentedly beside the stagnant waters of the desert, when, by dint of a little additional exertion, he might gain the border of the running stream.

And, indeed, the practitioner, who, from no loftier principle of conduct, feels anxious to possess himself of the knowledge with which this volume abounds, will, at least, be incited to the effort by the more powerful and almost unfailing spur of self-interest. No sooner is the medical attendant now-a-days summoned to the bed side of a gouty patient, elevated by the power of wealth or intellect above the lower classes of society (and to persons of the former description gout, for the most part, restricts its ravages) than he is interrogated respecting his acquaintance with the treatise of Dr. Scudamore. Nor enviable is the situation of that man, who is exposed to the humiliation of confessing his ignorance, and to the confusion excited in the mind of every one, not utterly destitute of delicate and exalted feeling, by the significant expressions of contempt to which such acknowledgment commonly gives rise. He must, at least, content himself with acquiring, in the opinion of his inquisitors, the reputation of a blockhead or a drone. Even we have, more than once, experienced no slight degree of astonishment in finding gentlemen, whose literary acquirements, we verily supposed, had never gone beyond the pale of the *New System of Cookery*, or the *Sporting Magazine*, more intimately acquainted with the opinions and practice of Dr. Scudamore, than ourselves.

On reverting to the *First Edition* of the work, which was published only one year previously to the appearance of the present (a tolerably sure criterion, by the bye, of the tone of public opinion respecting it), and on instituting a comparison between them, we shall be enabled more clearly to appreciate the leading differences which they exhibit, and the extent of addition which the latter has received.

The following are the Sections contained in the first Edition: *Gout; of Acute Gout; of Chronic Gout: of Retrocedent Gout; some Observations on Rheumatism;* and finally, *of Chronic Rheumatism.* The whole consists of four hundred and two pages.

In addition to these, the *present work* contains three new Sections, respectively entitled, *General Considerations on Morbid States of the Digestive Organs; on the Theory and Treatment of Gravel; and of Prophylactic Regimen.* There is, in the whole, an addition of one hundred and ninety pages; that is, on the subject of gout and rheumatism, a respective increase of sixty-five and thirty-three; while the three new sections occupy the remaining ninety-two. Not one line of these do we, on any acount, wish to see expunged. Yet is it, in our opinion, deeply to be regretted, at least for the sake of the numerous and less opulent practitioners by whom the first edition has been purchased, that the additional matter of the present was not published by Dr. Scudamore in the shape of an appendix. That surely is an erroneous system by which the expence, naturally attendant on the purchase of medical books, is, in any way, augmented; and their circulation and utility consequently restricted to a narrower sphere. In this light, we have ever beheld a succession of constantly enlarged editions of good works as a great and positive evil to the interests of the science with which they are connected. This is a hint to which the standard writers of the present day, and among these we hesitate not to class Dr. Scudamore, would, perhaps, do well to attend. Of the reptile tribe of scribbling knaves and empirics, who, at present, infest the metropolis with their puffs and calumnies, we do not speak. Were it not for the lamentable depravity of national taste and intellect, which the increased consumption of trash like this bespeaks, we should little care how frequently, or in how large a volume, these prolific animals gave out their literary spawn. The larger, the more inaccessible; and certainly in proportion to such inaccessibility, the less noxious.

The three new sections of Dr. Scudamore's work contain much valuable matter; and are quite equal in character and in style to those which constitute the other portion of the volume. We scarcely know how to pass upon them a more high or flattering compliment.

### Surgery.

### I.

*Practical Observations on the Treatment of the Diseases of the Prostate Gland, &c.* By Sir EVERARD HOME, Bart. &c. Vol. II. 8vo. pp. 301. Plates. London, 1818.

THE celebrity of the author, the titles with which his name is adorned, the size of the volume, and the important office which Sir Everard fills, all conspired to the anticipation of a rich feast in the perusal of the work under consideration. To say that we were disappointed is the cant of criticism; and we have not yet breathed enough of the courtly air, to eulogize a great name at the expense of self-respect. This piece of impolicy will doubtless be attributed to our rustic simplicity, or rather to our ignorance of the world; but, as we have hitherto found candour and honesty the best policy, in the long run, so we shall continue, *qualis ab incepto,* whatever may be the consequence.

We beg leave then to object to two large volumes on the prostate gland. To be useful to the student, or to the profession generally, a work should bear some proportion to the subject How many volumes should be written on the

*liver*, its functions, and its diseases, on Sir Everard's scale? Suppose an author published thirty volumes on the organ last mentioned; which thirty volumes are to be composed of *cases* of hepatic disease, (and a hundred volumes might easily be filled by any man in extensive practice) with a proportion of plates representing the organic changes which take place in the said viscus. The faculty would see, at a glance, that *details* were unnecessarily spread out, where *deductions*, occupying a small space, would have more effectually answered the object of promoting medical science. The thirty volumes, therefore, would not be as useful to the student and to the profession, as one small, but well arranged volume. Now we firmly believe, that if Sir Everard Home had compressed his two volumes into one very small one, omitting a display of unnecessary cases, and condensing his inferences, his deductions, and his rules of treatment, he would have conferred a lasting obligation on medical society, and his work would have descended, with honour, from generation to generation.

In the first chapter our author hazards an opinion,

"That the slow return of the blood from the neck of the bladder, arising from the disadvantageous situation of the veins respecting the heart, must, in advanced life, have a tendency to dilate them beyond their natural size; and, therefore, render an accumulation of blood, in these veins, more readily produced than in many other parts of the body."

He thinks that violent horse exercise often occasions rupture of veins in the prostate gland, and thus proves the principal cause of the disease. We should be more inclined to attribute the frequency of the disease to the unnatural degree of irritation, which, in the present licentious state of society, is kept up in the organs of generation by Cytherean excesses, and their attendants, strictures and bougies. The provocatives too, which sexagenarian lechers are so much in the habit of using, must tend to bring on this well deserved punishment of their ***hoary*** crimes.

In the third chapter, Sir Everard brings forward a symptom, attending enlargement of the "middle lobe," which had escaped him in the *first* volume, but which, he thinks, is of sufficient importance, in itself, to authorize the present publication. It is hæmorrhage, from sudden pressure, generally from equitation. Inflammation and ulceration of the "middle lobe," is a more frequent occurrence than our author before suspected.

"This, however, I do not believe forms any part of the progress of the disease, but must, *I am sorry to say*, be brought on by the *want of delicacy* in introducing the instrument into the bladder." P. 29.

We venerate Sir Everard Home more for this *honest and candid confession*, than for all the knowledge and skill displayed in the work before us. Infinite mischief is every day done by the rude and violent introduction of bougies, sounds, and catheters into the human urethra; but the misery which has been brought upon mankind, by *caustic*, is beyond belief. Were we disposed to publish a *case-book*, we could easily fill a volume with the pernicious effects of the "armed bougie;" *armed*, indeed, like the Indian's *kreese*, with a poison in its grooves, that leaves an irremediable wound!

In Chapter iv. Sect. 1. is related a rather interesting case, of hæmorrhage from the middle lobe, brought on by inordinate riding, in a gentleman, fifty-five years of age. Suppression of urine, and, indeed, a want of urinary secretion, supervened. He died, and was opened by Sir Everard. The middle lobe was found enlarged, spreading out unusually at the end; and on this was a projecting portion, where was discovered a ruptured vein filled with blood.

In the fifth chapter, Sir Everard informs us, that from his suggestions, Mr. Weisse (33, Strand) has been enabled to construct elastic gum catheters, so as to retain the curve at their points, when the stilette is withdrawn, and thus glide more easily into the bladder. We have examined these catheters, but have not yet had an opportunity of trying them; they appear adapted to their design. Sir Everard has also, in this chapter, pointed out a collar, by means of which the catheter may be secured in the bladder.

The sixth chapter is on the treatment of enlargement of the middle lobe; and considering that this is, or ought to be, the paramount subject of the volume, we found it extremely unsatisfactory.

"The tepid salt water hip bath" is condemned, and if any applications are to be made to the parts, "they should be such as produce cold." No vinegar or other acid, should be employed, lest the vapour passing from the mouth to the stomach, should act upon the neck of the bladder. No internal medicines, except senna and soluble tartar, are of use. If constipation be only in the lower bowels, glysters are serviceable; as two drachms of aloes dissolved in a pint of milk. When suppression of urine comes on, the elastic catheter, without the stilette, is to be cautiously introduced, and kept in the bladder till the urine begins to flow of a natural colour. It is dangerous to withdraw it, as the second introduction is often a work of difficulty. The means to be employed in preventing and assuaging the pain, are Dover's powder, in different quantities, according to the urgency of the symptoms, and opiate glysters. The remaining cases are merely illustrative of the foregoing rules.

The work concludes with a republication of Professor Brande's paper on Urinary Calculi, read before the Royal Society, May 19, 1808.

As fathers and mothers are generally too indulgent to, and proud of those of their children who are born in advanced life, so authors are often too partial to their latest productions. Milton, and his "Paradise Regained," are sufficiently illustrative of this remark. There is something melancholy, therefore, in beholding a man, who has deservedly attained eminence and celebrity, in his short span of existence, persevering in any course that may draw even the slightest shade over the splendour of former years. But honest advice or opinion is now, as it was three thousand years ago, a bitter and unpalatable potion, that nauseates the patient to whom it is given. Be it so! Let the unprincipled sycophant flatter and flourish; let the medical philsopher "be just, and fear not."

### II.

*Surgical Observations; being a Quarterly Report of Cases in Surgery, &c.* By Charles Bell. Part V.

When a book, emanating from a respectable source, is pronounced by a critical tribunal, to be totally useless, or void of information, we suspect there is something more than sound criticism at the bottom of the decision. On this account, Mr. Bell may have reason to complain; but, as "the blood of Douglas will defend itself," and as our object is, and will be, never to pollute the fair stream of science by personal allusions or altercations; so we shall endeavour to present our readers with an outline of the facts and opinions contained in the work before us, with an impartiality which is not on our lips, but in our breasts.

In the first Report Mr. Bell takes up the syphilitic question, and enters his caveat against the new practice of attempting the cure of that disease without mercury. His sentiments are so similar to our own, as expressed in the Periscope of our last number, that we do not deem it necessary to make any quotation from them here. We may state, however, that Mr. Bell ventures on the practice of bringing the system under the influence of mercury, even where the ulcer is spreading and eating deep, with a greenish yellow secretion, and dark slimy bottom. In our own practice, which has not been very limited, we have not found this treatment answer. We have enjoined quiescence, abstinence, purgation, leeches, and fomentations. These have stopped the spread of the ulcer, when mercury aggravated the symptoms.

Report II. *Injuries of the Urethra.* Under this head are related some cases of ruptured urethra, which are not intended for the upper classes of surgeons, but which exhibit a curious specimen of metropolitan ignorance in a practitioner. We think, however, that such latin as the following is not very creditable to the classic taste of our author. "*App. Hirudines octo ad imo abdomine.*" P. 34. We do not exhibit this for any other purpose but to prevent such carelessness in correcting the press.

In the Report shewing that "errors in digestion, and intestinal accumulations will produce pains in the bladder and urethra," we find much interesting matter, and many truths which we could corroborate by our own experience. Indeed, intestinal irritation propagates its influence so readily, even to the *remotest* parts of the system, that *neighbouring* organs ought always to be suspected of sharing in the evil.

Great mischief, we acknowledge with Mr. Bell, is every day done by the introduction of bougies, both by careless practitioners, and patients themselves, whereby the neck of the bladder, and the lacunæ of the urethra are irritated, when they offer resistance to the point of the instrument. We are convinced that many strictures are thus produced where they did not prevously exist.

The Report on *White Swelling* appears to us the most interesting of all. The great want of success which we so very generally experience in the treatment of this formidable complaint, renders us eager to embrace anyproposal, from a respectable source, for the mitigation of this affliction.

Mr. B. justly observes, that the various structures of the knee joint have all one common character of low vascularity, sensibility, and proneness to scrofulous inflammation. When this last has proceeded so far as to destroy the fine tissues of the articular surfaces, the spontaneous cure can only be expected from adhesion or anchylosis. Rest is indispensible towards this fortunate termination, and our author strenuously recommends a splint of tin, adapted to the thigh and leg, which shall prevent both the motion of the joint, and the distortion of the bones. To aid the proscess of adhesion or anchylosis, Mr. Bell entertained an idea of raising a new action by passing a seton directly through the diseased joint. This he actually performed in two instances. One will be a sufficient example.

" The young man's knee is swelled, puffy, and tender; the thigh and leg are considerably shrunk ; there is not much contraction or distortion, but the joint is weak, and unable to bear the slightest pressure. He is disturbed with frightful spasms, which make the limb spring up from the bed, wakening him in the night, and these are frequent and painful in the day also.

" I had a slender needle made, in length about twice the diameter of the knee. To this I attached a seton of six silk threads ; I passed it under the tendon of the patella, and across the joint. It gave no pain but in pricking the skin. I saw the limb put in junks before leaving the patient; I ordered eight leeches to be applied to the knee the same night, and impressed the patient with the necessity of keeping his bed, and not moving the limb." P. 85.

There was very little inflammation, but the cord broke short off when attempted to be drawn, and part of it remained in the joint, without injury.

Some time after this, Mr. B. passed another and a larger seton directly across the same joint, entering the needle betwixt the condyle and the head of the tibia, and bringing it out at the opposite point. From the ease with which it was carried through, Mr. B. supposes that the needle passed anterior to the crucial ligament. Some fever and pain followed this operation, but were reduced by leeches and cold lotions. This seton also remained fixed in the joint. The joint became anchylosed, and the man was able to walk home.

Two Reports follow on the Axis of the Pelvis, and on Hernia, in which are many useful observations. For the Remarks on Lithotomy we must refer to the work itself.

Mr. Bell's style is very singular, and we think far from good, or befitting a philosophical subject. But much of the matter is good, though common, and therefore it cannot be said, that

Materiam superabat opus.

---

### III.

*A Treatise on the Venereal Disease.* By JOHN HUNTER. *With an Introduction and Commentary,* by JOSEPH ADAMS, M. D. &c. &c. Second Edition, with Additions.

THE text of the illustrious Hunter stands the same, and the additional commentaries by his admirer and disciple, are not of sufficient importance to require notice. The title page of the work, however, excited in our minds many melancholy emotions. A ship is seen sailing on the vast ocean, under a press of canvas, followed, close behind, by a small sloop, as if in the act of pursuit. The former is John Hunter; the latter, Dr. Joseph Adams! Little did Dr. Adams think, when he planned this device, that, ere the page was dry from the press, his vessel, like that of his master, would sink to rise no more! The wave of oblivion has closed for ever over their material fabrics; but the spirit has fled to the source whence it emanated, and the archives of science will long record their names!

---

### IV.

*Observations on Hernia.* By CHARLES H. TODD, Member of the Royal College of Surgeons in Ireland, and one of the Senior Surgeons to the Richmond Surgical Hospital, &c.

[Dublin Hospital Reports.]

THIS is an important surgical document, and therefore we enrich the present department of our Journal by a pretty full analysis of Mr. Todd's paper.

During the last five years it has fallen to our author's lot to perform the operation for strangulated hernia thirteen times. The difference of structure, and density of the coverings of the hernial sac are not sufficiently attended to by young surgeons, though it is a matter of paramount importance.

"In the most common form of inguinal or scrotal hernia, we find, interposed between the integuments and sac, laminæ, formed of fasciæ, of the cremaster muscle, and of cellular membrane, more or less condensed, according to the duration, size, and previous management of the disease. Immediately under the integuments, the superficial or subcutaneous fascia constitutes a remarkable stratum, and none of the coverings of the sac exhibit so many different appearances as this. In some cases it will be found thick and dense, and consisting of several layers, between which blood-vessels and nerves ramify; while in others it will appear so thin and transparent as to be scarcely distinguishable from common cellular membrane." 228.

The next covering of the sac, in this kind of hernia, is derived from the edges of the external abdominal, united to the cremaster muscle and its subjacent cellular tissue. In old herniæ, this fascia is often very much thickened near the ring, forming strong fibrous bands across the neck of the tumour, which frequently compress the protruded viscus, and oppose its reduction. To the posterior surface of this the cremaster muscle adheres, the fibres of which are, in some instances, very much strengthened, while in others it is difficult to ascertain whether the muscle is anterior to the sac or not. In some instances, the tumour descends between the cremaster muscle and the chord; while in others, it will appear to have passed deeply within this sheath, or even behind or between the vessels composing the chord.

In those herniæ where the tumour escapes from the abdomen immediately behind the external ring, and descends immediately through that ring to the groin or scrotum, it has been affirmed, that the cremaster muscle is not expanded on the anterior surface of the sac, but retains its connexion with the common coverings of the chord. This, however, Mr. Todd has proved to be not uniformly the case.

"In a dissection of a double scrotal hernia which I made in January, 1804, where both protrusions were of this description, the cremaster muscle on one side was distinctly spread out on the fore-part of the sac, while at the other side, the character of the muscle was completely lost." P. 231.

In another instance, Mr. Todd found that a hernia, instead of passing anterior to the chord, protruded between the chord and the inferior pillar of the ring, so that the chord formed a sort of arch, embracing the neck of the sac for nearly two-thirds of its circumference, close to the external abdominal opening.

"Until this dissection, I had not conceived the possibility of the above occurrence. Now, however, on the strength of this case, in operating for the scrotal or inguinal hernia, I make it a rule, after laying open the superficial fascia, and before dividing the parts contiguous to the neck of the sac, to feel and minutely examine those parts, (particularly if they feel unusually bulky) and to satisfy myself as to the situation of the spermatic chord: having done this, I proceed with confidence to expose the superior pillar of the ring, which ought, in every instance, to be completely denuded before a bistoury is introduced to cut the stricture." 232.

The following critical observations on crural hernia are deserving of the surgeon's attention.

"The first important circumstance which generally attracts our attention on examining a crural hernia, is the peculiar situation which it occupies; being actually more on the abdomen than on the thigh, and ascending so much in front of the crural arch, and aponeurosis of the external oblique muscle, as strongly to resemble bubonocele. Although this fact has not been overlooked by modern writers on surgery, yet I have known practitioners of experience undecided as to the true nature of a hernia, until the integuments were divided, and the relative position of the neck of the sac and Poupart's ligament, brought into view.

"This position, occupied by the hernial tumour in almost every instance, has been thus accounted for by Mr. Lawrence, in his Treatise on Ruptures. 'Since the motion of the thigh and the more close adhesion of the integuments to the subjacent parts resist the increase of the tumour downwards, and the larger quantity of cellular and adipose substance at the bend of the limb offers less resistance, it comes forward to the surface, so as to lie in general in front of the crural arch.' (p. 349). Mr. Lawrence's explanation appears to me perfectly satisfactory; but when the student compares it with Mr. Cooper's description of the superficial fascia, he cannot subscribe to the accuracy of both. 'When the skin is removed from the crural arch and fore part of the thigh, (says Mr. Cooper) a thin fascia will be seen to extend over the tendon of the external oblique muscle, *and to adhere firmly to the edge of the arch, &c.*' Now, were this the case, it must be obvious, that as the superfacial fascia forms one of the coverings of the hernial sac, its connection with the crural arch would oppose the ascent of the tumour, and the increase of the swelling downwards would occur with much greater facility. A careful dissection of these parts, however, shews us, that the superficial fascia is really as loosely connected to the lower border of the obliquus externus, as to any of the other subjacent parts; and that when exposed, we can move it with our fingers on the parts over which it is expanded, with equal ease every where, except at the hollow of the thigh, where the cutaneous vessels and nerves, the lymphatics and their glands, are entangled with its laminæ.

"The fascia lata of the thigh, where it is intimately united to Poupart's ligament, sends off a thin but compact layer, which passes upwards closely connected to the aponeurosis of the external oblique muscle, by fine cellular tissue, and, like all other fasciæ, exhibiting varieties of structure in different subjects. This process of the fascia lata is generally overlooked in dissection, and when we dissect towards the thigh, is often raised with the superficial fascia, of which it then appears merely as a layer, firmly adhering to the crural arch; but, if we commence our dissection on the thigh, and trace the superficial fascia upwards, we shall raise it distinctly, and leave the deeper fascia in its situation.

"As the deep seated fascia of the abdomen is evidently continuous with the external surface of the fascia lata, it follows, that the hernia ascending in front of the crural arch, must be interposed between this and the superficial fascia; and consequently, that the close adhesion of the deep seated fascia to Poupart's ligament, cannot be an obstacle to the course of the hernial tumour upwards.*

"Both Mr. Cooper and Mr. Lawrence have expressed themselves strongly on the danger of mistaking a crural for an inguinal hernia, and have pointed out marks of distinction which require to be carefully attended to. These authors assert, that if the tumour, in femoral hernia, be drawn downwards, the crural arch may be traced passing over the neck of the sac; while, in bubonocele, it is found under that part. I have not succeeded satisfactorily in every instance, in deciding on the existence of a crural hernia by this rule; and I have observed, that it is not always practicable to draw the tumour downwards, particularly if inflammation has extended to the parts exterior to the sac; in endeavouring to draw the swelling towards the thigh, the integuments are rendered more or less tense, and this alone occasions a difficulty in feeling Poupart's ligament passing across the neck of the sac. A more unerring rule towards

---

* Scarpa erroneously describes the crural hernia, as being situated under this layer of the fascia lata.

"Below this glandulo-cellular layer, (the superficial fascia) another appears entirely aponeurotic, but of unequal density, that is, denser and firmer towards the ilium than in the vicinity of the pubis. This aponeurotic covering of the hernia, is only, in reality, the aponeurosis of the fascia lata which is extended upwards, and spreads over the femoral arch and inguinal ring, and in the male descends over the cremaster muscle. This aponeurotic web of the fascia lata, when it is about to pass over the femoral arch, adheres firmly to the Fallopian ligament, to which it is so firmly united, that it cannot be separated without laceration." P. 247.

a diagnosis, in my opinion, is to search for the neck of the tumour under the crural arch, and this is done with great facility in the flexed position in which the thigh is usually kept. In consequence of the relaxed state of the fascia lata, Poupart's ligament may be traced, extending from the superior spinous process of the ilium to the crural ring; but if the surgeon presses his fingers firmly at that part, between the hernia and the thigh, he will feel the neck of the tumour turning under the ligament, and then coming in front of it." In thin persons, the neck of the tumour can be distinctly felt in this situation; and in large women, in whom the integuments of the abdomen are generally loaded with fat, I have found this diagnostic mark more conclusive than that of Mr. Cooper."

Notwithstanding the length of the foregoing extract, we are tempted to introduce a still longer one, because we have tried, in vain, to condense it, and we are unwilling to pass it over.

"There are so many different opinions with respect to the seat of stricture in crural hernia, that I shall take the liberty of stating the result of my experience on this point. In five of the cases in which I operated, the intestine was effectually liberated by dividing the sharp aponeurotic edge which forms the pubal boundary of the femoral ring; and although many consider it difficult to introduce the finger so far into the neck of the sac as to feel this edge, and conceive that the division of a part of it is attended with considerable risk, yet the operations which I have performed have led me to think, that the stricture in this species of hernia is most commonly formed by the ring, and that it is removed with greater ease and safety by a cautious dilatation towards the pubis, than in any other direction.

"Mr. Cooper, amongst other objections which he has urged against the operation inwards, states, that if the hernia is large, this mode of dividing the stricture is not sufficient to allow of the return of the protruded parts, for it gives but little additional room, so that great force must be used to reduce the hernia.* This assertion of Mr. Cooper has not been confirmed by those cases and dissections which have fallen under my observation; in no instance have I found it necessary to cut more than a few fibres of what is called Gimbernat's ligament, and this has uniformly proved sufficient to enable me to return the protruded viscera without difficulty.

"An ample enlargement of the femoral ring inwards, appears to me quite practicable; nay, in the manner in which the operation is usually performed, Gimbernat's ligament may even be detached from the pubis to such an extent as to render the crural arch afterwards exceedingly weak, and consequently to expose the patient to larger visceral protrusions at this part.†

---

* See Cooper on Crural Hernia, p. 22.

† I was present at an operation once, in which this error was inadver-

" I operated for crural hernia, two years ago, on a woman in the Richmond Surgical Hospital; the stricture was removed by cutting a small portion of Gimbernat's ligament, and the contents of the sac, which consisted both of omentum and intestine, were easily reduced; however, the symptoms of strangulation continued, no discharge from the bowels could be procured, and she died on the following day.

" On dissection, it was found that the portion of intestine which had been protruded, had become entangled with the omentum after reduction, the latter having contracted a firm adhesion with the orifice of the hernial sac, and thus an internal strangulation took place, which proved fatal. On examining the crural ring, I ascertained that Gimbernat's ligament had been detached from the pectineal ridge for nearly half an inch, and that, had it been necessary, the enlargement of the ring might have been continued to a much greater extent in that direction.

" A few months ago I performed this operation on an old woman who had been brought into the Fever Hospital of the House of Industry, almost in a state of insensibility. She had had a strangulated hernia for several days. Her friends supposing she laboured under idiopathic fever only, obtained her admission into that part of the Institution; but the nature of her disease having been at once detected, she was placed under my care by Dr. Cheyne. On opening the hernial sac, the intestine was discovered dark-coloured, but apparently sound. Some slight adhesions existed between it and the sac; these I removed, and then endeavoured in vain to reduce the intestine.

" The border of Gimbernat's ligament was so distinct, that I availed myself of the opportunity of allowing an intelligent pupil, who assisted me in the operation, to feel it, and to compare its state of tension with the relaxation a small incision produced in it. On dividing the edge of the ligament, the intestine was returned without difficulty, and the patient recovered.

" The mode of dividing Gimbernat's ligament, which is usually adopted, and which has been recommended by Gimbernat himself, is, in my opinion, objectionable.

" In the first place, the crural ring is, in most cases, so completely filled up by the prolapsed viscera, that the introduction of a director is attended with considerable difficulty. Secondly, when both director and bistoury have been introduced, they are often so tightly embraced, that they cannot be very easily moved, and the operator, having both his hands employed in the management of instruments, cannot sufficiently guard the intestine from injury. And lastly, I have found, that the manœuvre of cutting the liga-

---

tantly committed, and where considerable embarrassment and delay occurred in consequence of the difficulty of retaining the intestines within the abdomen.

ment towards the spine of the pubis, at the same time that the surgeon is drawing out the instruments, is by no means a safe part of the operation, and requires much dexterity and experience.

"The division of the stricture in crural hernia, I think, is best effected by adopting the following rules.

"After the sac is opened, let the operator pass the forefinger of his left hand (if the hernia be on the right side, and *vice versa,*) as far as he can, without using force, into the neck of the sac, the back of the nail being turned towards the pubis, and its extremity being applied to the edge of Gimbernat's ligament; Mr. Cooper's bistoury* is then to be introduced, the finger serving as a conductor for it, the bistoury being held so, as that its edge shall look towards Poupart's ligament, and one of its sides be in contact with the nail of the conducting finger, between which and Gimbernat's ligament it is to be passed. Having ascertained with the blunt extremity of the bistoury, that it is fairly introduced into the crural ring, the intestine being protected, as much as possible, by the left hand of the operator, the edge of the bistoury is to be turned towards the pubis, and being pushed a little further into the ring, the sharp portion of its edge will be brought into contact with Gimbernat's ligament, and will divide a few fibres of it. The operator is still to keep the conducting finger between the intestine and bistoury, as thus he will be enabled to perceive the dilatation of the ring, and to judge of the extent to which it may be necessary to enlarge it."

This paper is equally creditable to Irish Surgeons and Irish Surgery.

---

### V.

*Remarks on Burns and Scalds, chiefly in Reference to the Principle of Treatment at the Time of their Infliction; suggested by a Perusal of the last Edition of an Essay on Burns, by* EDWARD KENTISH, M. D. &c. &c. &c. By NODES DICKENSON, of the Royal College of Surgeons; Staff Surgeon to H. M. Forces, &c. One Vol. Octavo, pp. 134. London, 1818.

OUR closing limits will not permit us to notice this little work to the extent which the importance of the subject deserves; and, indeed, had we ever so much space left, we

---

* This bistoury is delineated in Mr. Cooper's Work on Hernia. Plate XI. Part I.

find that it would be impossible to give any regular analysis of a work which is itself an *analysis*. Mr. Dickenson, who is an experienced and intelligent surgeon, has reviewed Mr. Kentish's work with much critical acumen, but in the most dignified and philosophical style. In this Review he has evinced a very creditable acquaintance with Medical Literature in general, and he has analysed the principles on which the efficacy or injury of the various applications may be explained, with great force, ingenuity and practical utility.

---

### VI.

*Minutes of Cases of Cancer, Part II. being further Reports of Cancerous Cases, successfully treated by the New Mode of Pressure, &c.* By SAMUEL YOUNG, Member of the College of Surgeons, &c. Octavo, pp. 280.

---

### VII.

*Commentaries on Cancer, Part II.* By WM. THOMAS, Member of the College, &c. Octavo. pp. 36.

THESE works need not detain us long. Mr. Young's *pressure* will succeed in no man's hands but those of the author; and therefore to the author we leave it, as doubtless he wishes it to be left.

Mr. Thomas's pamphlet is designed to recommend the internal and external use of arsenic in those stages of the disease where scirrhus is verging to the ulcerated state. But he lays down so many cautions, and so many undefined, and unintelligible rules, that it would almost appear that the work was similar in *design*, as well as execution, to Mr. Young's performance. We much doubt whether such books as these are calculated to advance the science, or immortalize the authors.

Non sic iter ad astra!

### PARTURIOLOGY.

### I.

### *Abstract of a Registry kept some Years in the Lying-In Hospital, Dublin.* By Joseph Clarke, M. D.

Independently of the talents and experience of the author, the report itself comes warmly recommended by its authenticity, fidelity, and the large sphere of practice whence it is drawn. We can only glance at the more prominent and useful features of this very interesting and modest Report.

1. Dr. Clarke strongly recommends us "to allow the uterus to gradually empty itself during delivery, first by expelling the head of the fœtus, and afterwards the shoulders and body by subsequent pains, with little or no aid." The circulation in the funis, he thinks, ought, in feeble infants, to be allowed to cease spontaneously before a ligature be applied. When the uterus shows a disposition to irregular contraction, the expulsion of the body of the child should be *retarded,* while a hand is kept on the abdomen, pursuing the fundus uteri till the fœtus is expelled, and pressure continued there in order to keep the womb contracted. A brisk purgative, given a few hours after delivery, has removed obstinate after-pains, when opiates and aromatics failed. Dr. C. thinks that extracting instruments are rarely necessary or safe.

"When labour is protracted to a dangerous length by *unusual resistance,* there is nothing but mischief to be expected from their application."—And again, "Let it be remembered, that in the hospital, such means were employed in one case out of 728; and in private practice, it is so long since I had occasion to use, or even think of using them, that I am persuaded a fair opportunity of applying forceps with good effect will not occur to a rational practitioner in one of a thousand cases." P. 372.

These are strong expressions, and from great authority. When a pelvis measures but three inches from pubes to sacrum, there will be a laborious labour; below this size it is scarcely possible for a *living* head to pass. It is of great importance to give a laxative medicine at the beginning of every labour likely to prove tedious, which may operate three or four times before the head becomes wedged in the pelvis.

2. In footling cases, the os uteri should be allowed to dilate slowly, and without interruption till it is completed. There is no necessity to interfere until the breech of the fœtus is expelled to the os externum. Breech presentations are always left to nature, except where the pelvis is defective. In cross presentations, Dr. Clarke has not seen a spontaneous evolution of the fœtus. One unequivocal instance of this occured in our neighbourhood within these few weeks. The arm was protruded; the child could not be turned; the contractions of the uterus at length forced down the doubled body; and lastly the breech was pressed down—the arm receded, and a safe delivery was effected. Is it not probable, that where turning cannot be easily accomplished, nature would in most cases, cause spontaneous evolution? Dr. C. queries—"would not a better chance be afforded to patients so situated, by perforating the thorax or abdomen so as to lessen their bulk?"

3. *Uterine hæmorrhage after* delivery only occurred in one case in a thousand. This our author attributes to the patients being kept very cool during labour, to the prevention of voluntary exercise during the dilation of the os uteri, and to the disuse of cordials.

4. *Convulsions.* Both in uterine hæmorrhage and convulsions, "one general principle directed our conduct in the treatment, viz. to trust to nature's efforts until the patient's life appeared to be endangered by the continuance of the disease, and then to interfere in the speediest and safest manner to effect delivery." When the forceps or lever could not be safely applied, the perforation and crotchet were used. Venesection and acrid glysters. Opium did no good. Insanity, of temporary duration, often follows repeated paroxysms of convulsions.

5. *Retention of the Placenta.* After waiting two hours, and using such gentle means as are commonly employed to promote its expulsion without effect, little is to be expected from the efforts of Nature, and the patient has the best chance of recovery from the prudent interposition of art."

The uterus ought to be tickled into action, rather than the secundines forcibly extracted. Much time should be employed, unless there be alarming hæmorrhage. Where a sand-glass contraction occasions the retention, then a *moderate* degree of force may be employed; if this does not succeed, give a large dose of opium, and renew your efforts when the anodyne has taken effect. When adhesion to the uterus is the cause, nothing can be advised, but to act according to circumstances. *Lacerations of the vagina, gangrene of the bladder,* &c. are of very rare occurrence, 1 in 2000.

"The only effectual mode of preventing such misfortunes is, by having early recourse to the perforator and crotchet. P. 389.

6. *Rupture of the Uterus,* Dr. C. thinks, is often confounded with laceration of the vagina. In the latter case, where the fœtus escapes into the abdomen, he thinks it better to extract it through the laceration, than have recourse to the operation of opening the abdomen. Eight cases of laceration of the vagina, and escape of the fœtus into the cavity of the abdomen are related. In some of them the child and placenta were easily delivered footling by the wound, but they all died.

---

### II.

*An Essay on the Symptoms, Causes, and Treatment of Inversio Uteri; with a History of the successful Extirpation of that Organ, during the Chronic Stage of the Disease.* By W. Newnham, Esq. Octavo, pp. 152, two plates, 1818.

Among the melancholy accidents to which the female is liable in the puerperal state, there are none more distressing than *inversi outeri.* Happily since the management of parturition has been taken out of the hands of ignorant midwives, and vested in those of the general practitioner, (a class which, as they are familiar with all forms of disease, so are they, in reality, the great depositaries of medico-chirurgical knowledge) this afflicting disease is but rarely to be seen. Yet its occurrence is still sufficiently frequent to entitle it to much consideration; and Mr. Newnham deserves thanks of his brethren for having detailed so clearly the interesting case that gave birth to the present volume; and for having, by much research, concentrated most of what has been said on the subject by British and Continental practitioners.

Mr. N. correctly states, that the most frequent cause of inversio uteri, is the rude attempt to hasten the expulsion of the placenta by pulling at the chord. It may also be occasioned by vehement efforts to bear down, on the part of the patient, at the moment when the fœtus is about to be evolved.

The immediate consequences of this accident are, *generally,* hæmorrhage; bearing down pains; syncope; convulsions; nausea; vomiting—death. If this first storm, however, is got through, the unhappy sufferer may fall a prey to consecutive inflammation and constitutional irritation, with gangrene of the part; retention of urine, &c. If the patient should have strength to struggle through this, she may happen to survive all these accidents, and recover a tolerable state of health, but still the uterus is liable to scirrhus or ulceration.

The inversio uteri may be easily reduced if it have taken place after delivery, and assistance immediately procured. We have had occasion ourselves to effect this in a poor woman whose uterus was inverted by a drunken midwife. We resided within a few yards of the spot, and by laying the head low, and making steady and gentle pressure on the fundus, we re-inverted and replaced the organ in about two minutes. The woman ultimately did well.

When some time has elapsed, we can easily conceive that the reduction will be most difficult, if not impossible, owing to the contraction of the os uteri above the tumour prohibiting its re-entrance into the pelvis.

> "It is here the wisest and best practice to introduce the hand, and gently grasping the tumour, endeavour to re-invert the uterus, by returning *first* that portion of it, which was *last expelled* from the os uteri. This process will be considerably assisted by pressing upwards the fundus of the uterus, at the same time that we compress gently its superior portion." P. 16.

Here, indeed, the rules of taxis in strangulated hernia pretty nearly apply. Dr. Denman found the reduction impracticable after a lapse of four hours.

Under the head of "Chronic inversion of the uterus," Mr. Newnham makes many judicious remarks; and the subject naturally turns to the propriety of extirpating a part which is a constant source of irritation to the system, and of misery to the patient. The late proceedings of Dupuytren and Osiander assure us, that considerable portions of the uterus may be cut away without much danger or difficulty. These surgeons have operated several times, and no alarming hæmorrhage or other unpleasant symptoms have supervened. In several instances, it is true, the disease has returned, or attacked other parts of the body; but unfortunately this stigma attaches to operations for scirrhus in most other parts of the body. We shall now proceed to give a condensed outline of the case which gave origin to the volume under review.

Mrs. Glasscock, ætat. 24, consulted our author in April 1817, on account of a constant mucous discharge from the vagina, accompanied by frequent hæmorrhage. The patient's countenance was exanguious, her appetite declining, and she was subject to frequent and prolonged fits of syncope.

"On examination, I discovered, in the vagina, a tumour of considerable size; somewhat of a pyriform shape, larger at its base than at its superior extremity; but not attached by a very narrow neck; surrounded at its apex by the os uteri, between which and the tumour, the finger could be readily passed without discovering any immediate connexion, as far as I could ascertain, nearly insensible; and which had never occasioned pain." P. 32.

This patient had been delivered on the 21st of January 1817, of her first child, after a natural labour. The funis was remarkably short, from her own account; the placenta adherent; and hæmorrhage had succeeded its removal. Dr. Davis saw her on the 10th day after delivery, and found the tumour in question, which he appears to have considered *inversio uteri.*

On the 7th of April 1817, Mr. N. applied a ligature round the cervix of the tumour; which, after being tightened more or less frequently till the 2d of May, on that day produced a separation of an inverted uterus. The tumour was about the size of a human heart.

"The partial adhesion of its surfaces, where they had been acted on by the ligature, renders the demonstration of the regular uterine interior somewhat confused; there is, however, a defined cavity in its centre, lined with a membrane, which is clearly peritoneum, on the sides of which may be distinctly traced the broad ligaments. On making an incision into its substance, the muscularity of its structure is clearly perceptible." P. 49.

The patient convalesced, and in five weeks after the removal of the tumour, she returned to town perfectly well. Her breasts are plump, and she enjoys the connubial rites as well as ever.

Under the heads of "Uncertainty of Diagnosis," "Consequences of Inversio Uteri," "General Prognosis and Means of Relief," and "Extirpation of the Uterus, &c." Mr. N. has made many excellent observations, and collected from various sources the sentiments of the best authors on these subjects. He has evinced very considerable erudition and research; and supports, with honour, the respectability of that rising class of practitioners, the Surgeon-Apothecaries. Fully convinced of the national importance of this class, we shall ever hail, with gladness, any thing from their pens, and contribute all in our power to stimulate them to the record of facts, and the diffusion of that practical knowledge which they unquestionably possess.

---

